# Experimental Characterization and Modeling of High Hole Mobility GeSn Quantum Wells: The Role of Alloy Disorder Scattering

**DOI:** 10.1002/smsc.202500589

**Published:** 2026-03-31

**Authors:** Troy A. Hutchins‐Delgado, Siddhant Gangwal, Steven Akwabli, Adelaide Bradicich, Priyanka Petluru, Hryhorii Stanchu, Sudip Acharya, Robin Scott, Nick Rosson, Michael Povolotskyi, Chia‐Tse Tai, Chia‐You Liu, Jiun‐Yun Li, Michael P. Lilly, Winson C. H. Kuo, Stephen D. House, Dragica Vasileska, Shui‐Qing Yu, Tzu‐Ming Lu

**Affiliations:** ^1^ Center for Integrated Nanotechnologies Sandia National Laboratories Albuquerque USA; ^2^ School of Electrical, Computer and Energy Engineering Arizona State University Tempe USA; ^3^ Department of Electrical Engineering and Computer Science University of Arkansas Fayetteville USA; ^4^ Lawrence Semiconductor Research Laboratory, Inc. Tempe USA; ^5^ Amentum Hanover maryland USA; ^6^ Graduate Institute of Electronics Engineering National Taiwan University Taipei Taiwan; ^7^ Center for Integrated Nanotechnologies Los Alamos National Laboratory Los Alamos USA

**Keywords:** alloy disorder scattering, GeSn quantum wells, high mobility, modeling and simulation, quantum hall

## Abstract

Understanding mechanisms influencing electrical transport in material systems not only provides a scientific explanation for observed behavior but also offers insight into ways to enhance transport in devices. This study reports experimental hole mobility of $8\times 10^{4}\;{\mathrm{cm}}^{2}\;V^{-1}\;s^{-1}$ in a Ge_0.92_Sn_0.08_, the highest recorded mobility for the GeSn system. A study of the material's quality is presented using structural and electrical characterization techniques, with transport data being supported by simulations using an extensive modeling framework. Quantum Hall measurements further indicate the material's high quality and potential spintronic applications, with extracted values of $0.0689m_{0}$ and 13.6 for the effective mass and effective g‐factor, respectively. It is observed that transport is limited by alloy disorder scattering at cryogenic temperatures. A comparative study between the presented structure and similar quantum well heterostructures revealed that the difference in hole mobilities is captured by a disparity in the reduced nominal alloy disorder scattering potential ($\Delta U_{\mathrm{alloy}}$ = 0.8 eV), that is lower than the value of a fully random alloy ($\Delta U_{\mathrm{alloy}}$ = 1.4–1.7 eV) potential. The difference in $\Delta U_{\mathrm{alloy}}$ suggests that heterostructures with similar geometries and alloy compositions can have different alloy disorder scattering, implying that an underlying mechanism, such as short‐range order, may be responsible and warrants further investigation.

## Graphical abstract

This study reports record‐high hole mobility in undoped GeSn quantum wells, validated through magnetotransport measurements. By modeling the transport properties, the authors discover that alloy scattering in their material is significantly weaker than predicted for random alloys and previously studied materials. The possibility of attaining ultrahigh hole mobility highlights GeSn's potential for high‐speed electronic and quantum computing applications.

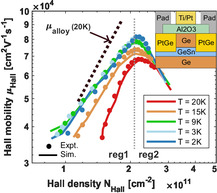

## 1 Introduction

Silicon (Si) and Si‐rich materials have dominated the microelectronics industry; however, the physical limitations of CMOS scaling have inspired scientific research to explore alternative material systems that can be effectively integrated into the existing Si infrastructure. This has led to increased interest in exploring materials in the group‐IV alloy system (SiGe, SiGeSn, GeSn, SiSn, etc.), as they are isoelectronic with Si. With improved material quality, group‐IV heterostructures show great promise not only for electronic applications, but also photonic [1, 2], spintronic [3, 4, 5], thermoelectric [6], quantum photonic [7],and gate‐defined quantum dots scaled to large arrays [8, 9] for quantum processors [10].

Analogous to the high electron mobilities in Si‐rich Si/SiGe quantum wells [11, 12], Ge‐rich Ge/SiGe quantum wells have demonstrated hole mobilities exceeding $4\times 10^{6}\;{\mathrm{cm}}^{2}\;V^{-1}\;s^{-1}$ [13]. High mobilities and strong spin–orbit coupling (SOC) in holes in Ge make Ge‐rich heterostructures a strong candidate for hole, spin qubits [14]. Ge is an indirect bandgap semiconductor with a small energy difference between the direct and indirect bandgaps. Consequently, electrons experience strong intervalley scattering, leading to longer lifetimes. There is also an effort to engineer the band structure of Ge by strain and alloying. It is well known that strain increases the hole mobility in Ge. It is also shown that alloying with Sn lowers the direct bandgap in Ge, such that GeSn alloys demonstrate a direct bandgap beyond a particular Sn concentration [15, 16]. As a result, Ge‐rich heterostructures have gathered interest for optoelectronic applications like infrared photodetectors [17, 18, 19] and electrically injected lasers [20, 21, 22]. While GeSn is widely studied for photonics, its potential for high‐speed logic and quantum computing relies critically on minimizing disorder‐induced scattering, which remains a primary bottleneck compared to other material platforms.

In this study, we focus on Ge‐rich GeSn alloys as their low cost, efficiency, and tunability of bandgap make them strong candidates for the development of next‐generation technologies. Theoretical studies of GeSn (and Si–Ge–Sn) alloys have predicted the presence of short‐range ordering (SRO), in contrast to the conventional treatment of alloys as random solid solutions [23, 24], which leads to changes to electronic band structure that can be used to enhance transport characteristics. The presence and degree of SRO can be ascertained via experimental techniques like atomic probe tomography (APT) [25] and extended X‐ray absorption fine structure (EXAFS) [26], [27]. Subtle variations in the electronic band structure, due to strain, alloying, and possible SRO, ultimately influence the electrical properties of the heterostructures. Thus, to unlock the full potential of GeSn devices, it is important to understand how the electronic transport is influenced by these phenomena.

Our study includes the investigation of correlations between material structure and electronic transport in shallow and undoped GeSn/Ge heterostructures grown by Lawrence Semiconductor Research Laboratory, a commercial SiGeSn epitaxy supplier. We report a record‐high hole mobility of $8\times 10^{4}\;{\mathrm{cm}}^{2}\;V^{-1}\;s^{-1}$ at 2 K, which is a fourfold improvement over similar materials [28, 29]. We comprehensively investigate the overall quality of the material structure, measure electrostatic carrier properties through quantum Hall measurements and carrier densities, and provide calculations to help explain the unprecedentedly high carrier mobilities. The theoretical study includes mathematical modeling of device electrical properties using self‐consistent Schrödinger–Poisson (SCSP) formalism [30] and two‐dimensional Monte Carlo (2DMC) techniques. The models are supported by ab initio calculations using density functional theory (DFT) to calculate the electronic band structure and extract critical material parameters for the alloy system. Supported by a thorough comparison with the previously reported state‐of‐the‐art GeSn sample data [28], we aim to (1) understand and explain the physical reasoning behind the high hole mobilities and (2) predict the theoretically possible values for obtainable hole mobilities in GeSn quantum wells.

## 2 Material Characterization

**FIGURE 1:**
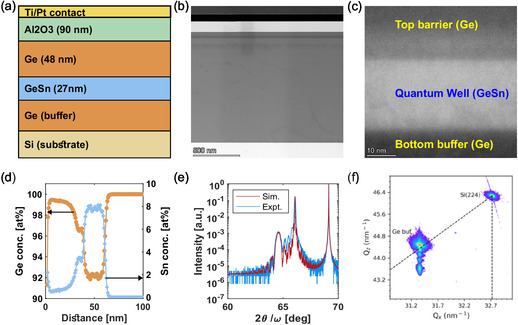
Structural characterization: (a) schematic of the material stack. (b) Low‐magnification BF STEM image. (c) HAADF STEM image (d) Ge concentration (orange) and Sn concentration (blue) as a function of depth obtained by SIMS. (e) Comparison of 2*θ* − *ω* XRD data. (f) RSM of the structure. BF STEM = Bright‐field scanning transmission electron microscopy; HAADF = high‐magnification high‐angle annular dark field; RSM = reciprocal space mapping; SIMS = secondary ion mass spectrometry; XRD = X‐ray diffraction.

**FIGURE 2:**
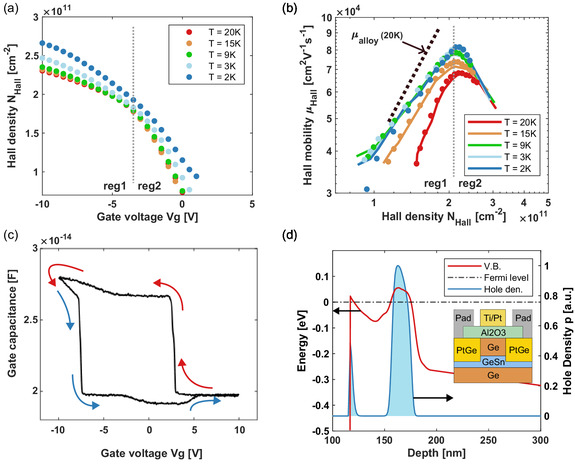
Low‐temperature transport characterization: (a) Hall density vs. gate voltage at 2 K (blue), 3 K (light blue), 9 K (green), 15 K (orange), and 20 K (red). Region reg1 indicates single‐channel transport through the quantum well, and region reg2 indicates two‐channel transport via the quantum well and the surface channel. (b) Hall mobility vs. Hall density. Here, markers indicate experimental data and the lines are from simulations (c) Capacitance vs gate–voltage at 2 K going from positive bias to negative bias (red) and negative bias to positive bias (blue). The mobility limit of alloy disorder scattering (at 20 K) is shown in a dotted black line. (d) Variation of valence band energy and hole densities (a.u.) at −5 V (near the formation of surface channel) along the depth of the device. The Hall bar device is shown in the inset.

GeSn/Ge hole quantum well heterostructures were grown on 200 mm (100) Si wafers by Lawrence Semiconductor Research Laboratories using previously reported techniques for Ge/SiGe [31] and GeSn/Ge [28, 32] growth. Figure 1a shows a schematic of the structure starting with a 1100 nm relaxed Ge buffer atop the silicon substrate, providing a virtual substrate for the compressively strained 27 nm ${\mathrm{Ge}}_{1-x}$Sn*_x_*, *x* = 0.08 (or 8%) quantum well. A 48 nm Ge layer is grown on top of the GeSn hole quantum well, providing a top barrier. A low‐magnification bright‐field scanning transmission electron microscopy (BF STEM) image in Figure 2b displays the material stack and shows that the Ge buffer pins dislocations far from the quantum well, in the Si substrate and Ge buffer region. It can be seen that the majority of dislocations are present at the Si–Ge interface.

Secondary ion mass spectrometry (SIMS) was performed to provide spatial mapping of the Sn concentration (shown in Figure 1d). The SIMS analysis reveals that while the transition from the bottom Ge buffer to the GeSn quantum well is abrupt (1.51% ${\mathrm{nm}}^{-1}$), the transition from the GeSn quantum well to the Ge top barrier is not. The SIMS data show that the transition from an 8% Sn to about 3.5% is linear (1.07% ${\mathrm{nm}}^{-1}$), followed by a nonlinear grading down to about 0.5%. Contrast from high‐magnification high‐angle annular dark field (HAADF) STEM mirrors the SIMS data as shown in Figure 1c. The bottom Ge buffer has the darkest contrast, indicating the lowest Sn concentration, and has a sharp interface with the brightest contrast Sn concentration of the quantum well. The quantum well and top barrier interface is a sharp indicator of the material's high quality, but the top barrier has an immediate, intermediate contrast while grading to a darker contrast further from the quantum well.

X‐ray diffraction (XRD) analysis was performed using a 2θ−ω scan. The XRD results, shown in Figure 1e, show excellent agreement between the experimental and simulated data assuming a 104% overrelaxed Ge buffer, a strained GeSn QW with 8% Sn, and a linear distribution of Sn concentration from 2.5% to 0% in the top barrier. These values were determined by approximating energy‐dispersive spectroscopy (EDS) data (not shown) to a smoothed model, performing reciprocal space mapping (RSM), and optimizing over the fit Sn concentration in the quantum well. Reciprocal space maps were obtained to measure the in‐plane and out‐of‐plane lattice constants of the stack constituents (Figure 1f). The Si (224) peak appears at $\left(Q_{x},Q_{z})=(32.72,46.30\right)$. Originating from this point, we indicate the 100% relaxation path (dashed line) where the in‐plane and out‐of‐plane lattice constants are equivalent. The Ge (224) peak appears above this line at $\left(Q_{x},Q_{z})=(31.36,44.50\right)$, indicating that the Ge is overrelaxed (*R* = 104%). We observe the GeSn (224) reflection vertically aligned with the Ge reflection, indicating that the GeSn is coherently strained to the Ge buffer. Altogether, these results indicate good crystalline quality while quantifying the composition profiles, layer thicknesses, and strain.

## 3 Results and Discussion

This section is subdivided into three broad subsections as follows. In Section 3.1, we discuss the results of various experiments performed to determine the electrical properties of the material system. This includes extracting hole densities ($N_{\mathrm{Hall}}$), Hall mobilities ($\mu_{\mathrm{Hall}}$), and device capacitances. We include an extensive theoretical analysis to provide a deeper understanding of the physical phenomena responsible for the observed quantities. In Section 3.2), we discuss the results of quantum Hall measurements and Shubnikov–de Haas effects, which are important for the characterization of 2D (and quasi‐2D) systems. In Section 3.3, we compare the presented results with results from the literature for *nominally similar* devices. Using a rigorous Monte Carlo model, we discuss the role of alloy disorder scattering and how it limits the hole mobility in the GeSn system.

### 3.1 Hall Bar and Capacitance–Voltage Measurements

Low‐temperature Hall and capacitance–voltage measurements were performed in a closed‐cycle 1 K cryostat using standard low‐frequency lock‐in techniques. Effective Hall densities, $N_{\mathrm{Hall}}={\left[e,\cdot ,R_{H}\right]}^{-1}$, are determined by linearly fitting measured transverse resistivity ($\rho_{xx}$) vs. magnetic field strength (B), to extract the Hall coefficient ($R_{H}$) as the slope of the fit. We determine the effective Hall mobilities, $\mu_{\mathrm{Hall}}={\left[e,\cdot ,N_{\mathrm{Hall}},\cdot ,\rho_{xx}\right]}^{-1}$, from $N_{\mathrm{Hall}}$ and $\rho_{xx}$ at B=0. The effective Hall densities and mobilities are equal to the actual carrier densities and mobilities in the case of a single transport channel. However, in the case of two transport channels, the effective Hall densities and mobilities are calculated as,

(1) $$N_{\mathrm{Hall}}=\frac{{\left(N_{1}\mu_{1}+N_{2}\mu_{2}\right)}^{2}}{N_{1}\mu_{1}^{2}+N_{2}\mu_{2}^{2}}$$

(2) $$\mu_{\mathrm{Hall}}=\frac{N_{1}\mu_{1}^{2}+N_{2}\mu_{2}^{2}}{N_{1}\mu_{1}+N_{2}\mu_{2}}$$

Hall measurements, shown in Figure 2a,b, were performed as a function of gate bias and at several temperatures: 2 K (blue), 3 K (light blue), 9 K (green), 15 K (orange), 20 K (red). The large Hall density range of about $2.5\times 10^{10}-25\times 10^{10}\;{\mathrm{cm}}^{-2}$ validates the high structural quality of the heterostructure and demonstrates the tunability of transport in this system. Furthermore, the Hall density versus gate bias data (Figure 2a) shows a change in slope at about −4 V in region reg1, without approaching a plateau, suggesting that at this gate voltage a second channel turns on and contributes to the hole transport. The formation of the second channel (at about −4 V) is confirmed by the SCSP modeling of the electrostatics.

This qualitative behavior is well known for enhancement‐mode buried quantum well transistors and has recently been demonstrated in high‐mobility Ge/SiGe double quantum well heterostructures [31, 33]. The hole transport in the second channel in this structure further supports the claim of the material's high quality and a clean oxide–semiconductor interface. In this context, the quality of the interface reflects the low concentration of surface defects. High density of defects inhibits electrical transport for both holes and electrons alike. A measured transport due to the channel formed at the interface (for high, negative bias) indicates a low concentration of surface defects, and thus, a higher quality of interface. The temperature dependence of the density–voltage relation is negligible for temperatures lower than 9 K. At temperatures greater than 9K, we observe a greater impact of temperature variation in the low and high density extremes.

The mobility–density data in Figure 2b show a Hall mobility starting around $3\times 10^{4}\;{\mathrm{cm}}^{2}\;V^{-1}\;s^{-1}$ at the conduction threshold and increasing up to about $8\times 10^{4}\;{\mathrm{cm}}^{2}\;V^{-1}\;s^{-1}$ at a density $2\times 10^{11}\;{\mathrm{cm}}^{-2}$. The peak mobility of $8\times 10^{4}\;{\mathrm{cm}}^{2}\;V^{-1}\;s^{-1}$ is the highest hole mobility achieved in a GeSn system to date. The simulated data (represented by solid lines) are within 95% accuracy with respect to the experimentally measured hole mobilities (represented by solid dots). Once again, the hole transport can be roughly split into two regions of operation. In reg1, a single channel is present and confined in the GeSn quantum well. As the applied bias increases, reg2, a parallel channel is formed at the ${\mathrm{Al}}_{2}O_{3}$/Ge interface. In semiconductors, electrical transport is inhibited by various physical phenomena, which are explained via different scattering mechanisms. For example, coulomb scattering for carrier–ion interactions, acoustic and optical phonon scattering for carrier–phonon interactions, piezoelectric scattering, etc. [34]. To leverage the potential of GeSn systems, it is important to understand the reasons for the observed hole mobilities. It is crucial to investigate the scattering mechanisms responsible for influencing mobility in this GeSn channel.

The solution of the 1D SCSP problem (Figure 2d) shows that the carrier wavefunctions are localized within the two channels and do not overlap at low temperatures. This indicates independent electrical transport in the two “individual” channels. The transport characteristics for a quasi‐two‐dimensional system are obtained by solving the Boltzmann transport equation (BTE) for carriers confined in different subbands [34, 35, 36]. For our system, we simulate the low‐temperature hole transport characteristics for carriers residing in the subbands corresponding to the heavy‐hole (HH), light‐hole (LH), and split‐off (SO) bands. Therefore, dominant scattering mechanisms at low temperatures in the GeSn quantum well are alloy disorder scattering and, to some extent, acoustic and nonpolar optical phonon scattering. For the carriers residing in the surface channel formed at the ${\mathrm{Al}}_{2}O_{3}$ and the Ge heterointerface, surface/interface roughness and phonon scattering due to acoustic and nonpolar optical phonons limit the low‐field mobility. The reduction in hole mobility values at higher sheet carrier densities (reg2) is attributed to the formation of the second channel at the ${\mathrm{Al}}_{2}O_{3}$/Ge interface. This channel experiences strong interface roughness scattering. However, the carrier densities observed were not sufficiently large to observe the expected temperature‐dependent variation, which results in observed overlap of the mobility curves for different temperatures. In reg1, the mobility limited by surface roughness scattering is estimated around $1.2\times 10^{6}\;{\mathrm{cm}}^{2}\;V^{-1}\;s^{-1}$ and thus plays an insignificant role. Subsequently, the hole mobility is limited by alloy disorder scattering in the low‐density regime, making it imperative to further investigate the impact of alloy disorder scattering on the structures. A detailed discussion on alloy disorder scattering is presented in Section 3.3, and thorough details about the various scattering mechanisms incorporated in our transport model are given in the Section 5.

Capacitance–voltage measurements (shown in Figure 2c) were performed to provide additional insight into how charges behave spatially with respect to gate bias. To perform the measurements, the Ohmic contacts of the Hall bar device were all tied together while the gate was biased using a DC + 10 mV, 1 kHz AC signal provided by an arbitrary waveform generator. The measurements used the same 1 K system as the Hall measurement and used standard lock‐in based techniques. The data include a capacitance offset due to the overlap of the gate and Ohmic contacts and a parasitic capacitance from the wiring infrastructure. However, these two capacitances do not change the qualitative behavior observed. Figure 2c shows the capacitance–voltage characteristics of the device, starting at an applied bias of 10 V. At this bias, the device is depleted of holes. As the bias is lowered (indicated by red arrows), holes accumulate in the quantum well region, leading to a rise in capacitance. The hole charge density saturates at about 3 V, not changing as bias is further lowered. Around −4 V, the capacitance starts to increase again, indicating the accumulation of charge at the oxide–semiconductor interface channel. At a bias of −10 V, the voltage is then swept in the positive direction (indicated by blue arrows), and the capacitance decreases, indicating depletion of holes from the oxide–semiconductor interface channel. At about −7 V, the sharp drop in capacitance indicates the depletion of holes from the quantum well region as well. There is a further drop in capacitance when increasing the gate bias to about 0 V, marking a true minimum in the capacitance and indicating the material is fully depleted. This last drop is due to the emptying of interface traps. The interface traps are responsible for the large hysteresis displayed in the capacitance–voltage data. Continuing to increase the bias, an increase in capacitance is observed just before 5 V, whereby the capacitance overlaps with the negative sweep minimum. This increase in capacitance, therefore, suggests that inversion is occurring and accumulating electrons, likely filling traps at the oxide–semiconductor interface, due to the capacitance in this regime being equal to the value in the regime with hole traps. This is not observed in the transport measurement because the contacts are PtGe, which form a large Schottky barrier for electrons due to Fermi level pinning [4, 37].

The transport and capacitance–voltage experiments show the formation of a second channel at the oxide–semiconductor interface when the device is biased at large negative gate voltages. The SCSP simulations support this conclusion. This can be explained by understanding the behavior of the valence band with respect to the Fermi level ($E_{f}$) for the device (shown in the inset of Figure 2d). When negative gate biases are applied, the valence band is pulled up (bends upwards) and holes accumulate in the GeSn quantum well. As the applied bias increases (becomes more negative), the valence band is pulled further, leading to the accumulation of the holes near the ${\mathrm{Al}}_{2}O_{3}$/Ge interface, and the inevitable filling of surface trap states at the ${\mathrm{Al}}_{2}O_{3}$/Ge interface. High density of traps, along with electrostatic screening due to accumulated charges, makes it difficult for carriers to move in the region. This behavior is generally observed in conventional quantum well heterostructure field effect transistors due to highly disordered oxide–semiconductor interfaces. However, transport contributing surface channels have been observed in high‐quality Si/SiGe quantum well heterostructures due to a good SiO_2_/Si interface [38]. In this study, we observe that the quality of ${\mathrm{Al}}_{2}O_{3}$/Ge interface can support hole transport in the surface channel. The behavior of this surface channel is similar to that of Ge MOSFETS reported previously [37].

### 3.2 Quantum Hall Measurements

**FIGURE 3:**
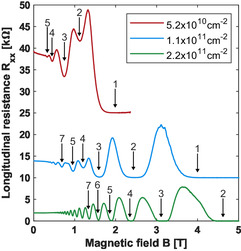
Longitudinal resistance vs. magnetic field at 1 K for different Hall densities. The blue curve is offset by 10 kΩ and the red curve is offset by 25 kΩ for clarity.

**FIGURE 4:**
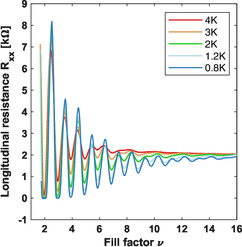
Longitudinal resistance vs. fill factor at different temperatures for the Hall density of $2.2\times 10^{11}\;{\mathrm{cm}}^{-2}$.

**FIGURE 5:**
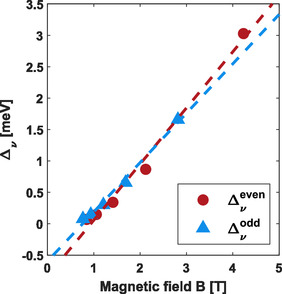
Fill‐factor thermal excitation gap vs magnetic field at a Hall density of $2.2\times 10^{11}\;{\mathrm{cm}}^{-2}$. The linear fits are used to extract an effective g‐factor of 14.57 and an effective mass of $0.0685m_{0}$.

Further reducing the temperature and increasing the magnetic field allows us to probe the quantum Hall regime, whereby Shubnikov–de Haas oscillations provide further insight into the material's quality and allow extracting parameters such as the effective mass and effective g‐factor. The extracted effective mass was then used in the simulation of the transport. Quantum Hall measurements were performed using a dilution refrigerator with a base temperature of 30 mK. Shubnikov–de Haas oscillations in the longitudinal resistance, $R_{xx}$, were observed at three different densities that sample the entire density range as shown in Figure 3. The lowest density of $5.2\times 10^{10}\;{\mathrm{cm}}^{-2}$ (red) shows the seven lowest Landau levels, with only the five lowest clearly visible in the plot. The odd fill factors are dominant, although no splitting limit is observed where only the odd fill factors remain. The mid density of $1.1\times 10^{11}\;{\mathrm{cm}}^{-2}$ (blue) shows oscillations for fifteen Landau levels, although only the lowest seven are labeled here. The splitting limit is observed around 0.8 T in which only odd fill factors remain at lower magnetic fields. The data for the highest density of $2.2\times 10^{11}\;{\mathrm{cm}}^{-2}$ (green) show about 20 Landau levels (with only the lowest 7 labeled). There is no visible spin splitting limit observed in the oscillations. The absence of a splitting limit with many oscillations observed suggests that the Landau levels are equally spaced due to the effective g‐factor and effective mass ($m^{*}$) product approximately equal to unity. The temperature dependence of the oscillations was investigated in a large range from 30 mK up to 10 K, but only the highest density was robust enough to obtain confident fits, extracting thermal excitation gaps. The temperature dependence of $R_{xx}$ versus fill factor ν is shown in Figure 4. The thermal excitation gaps as a function of magnetic field (Figure 5) then allow extraction of the effective g‐factor and effective mass from the odd and even thermal excitation gaps, respectively [39]. An effective g‐factor of 13.6±1.2 and an effective mass of $0.0689m_{0}\pm 0.0048$ were obtained from linear fits, whereby the errors are the standard deviations calculated from the 95% confidence intervals with the effective g‐factor being directly calculated and the effective mass calculated in quadrature. Additional details about the analysis can be found in the Supporting Information (SI). The effective mass value is consistent with unreported values from previous collaborators [28], recent results [29], and is similar to what has been observed in Ge/SiGe quantum well heterostructures [13, 39, 40, 41]. Likewise, the large effective g‐factor is consistent with recent results in GeSn/Ge [29] and reported values in Ge/SiGe quantum well heterostructures [13, 39].

### 3.3 Theoretical Investigation of the Hole Mobility

**FIGURE 6:**
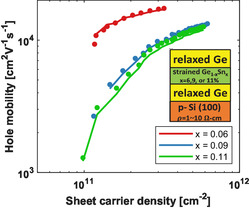
A theoretical study of previously reported hole mobilities for similarly grown heterostructures [28]. *Data reproduced with permission*. The dots represent experimental data, and the solid lines represent simulated data for structures with 6% Sn (red), 9% Sn (blue), and 11% Sn (green) compositions.

As mentioned briefly before, we investigated the hole transport in previous state‐of‐the‐art high‐mobility GeSn/Ge heterostructures. It is also established that in the low‐density regime (reg1), where the carriers are confined in a single channel in the GeSn quantum well, the hole mobility is limited by alloy disorder scattering. A comparative summary of previously reported mobility values in GeSn quantum wells is shown in Table 1, out of which we investigated the hole transport reported in [28]. As seen from the inset of Figure 6, these are nominally identical GeSn/Ge structures (as shown by the structural cross section inset) with the following concentrations of Sn: 6% (red), 9% (blue), and 11% (green). They have a 500 nm bottom Ge buffer, 10 nm compressively strained Ge${1-}_{x}$Sn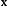 quantum well, and a 23 nm top Ge buffer. While the layer thicknesses in these structures are slightly different from ours, the simulations account for the effect of these, as explained in Section 5 (Methods section). We obtained a good agreement (±10%) between our 2DMC simulations and the experimentally measured values for the hole mobilities, as shown in Figure 6.

**TABLE 1:** Transport comparison for different GeSn QW structures.

% Sn	Barrier/buffer	$t_{\mathrm{top}}$, nm	$t_{\mathrm{qw}}$, nm	$t_{\mathrm{bot}}$, nm	Density range, $10^{11}\;{\mathrm{cm}}^{-2}$	Mobility range, $10^{3}\;{\mathrm{cm}}^{2}\;V^{-1}\;s^{-1}$	Temperature, K	Ref
6	Ge	23	10	500	1.0–3.3	4.0–20	1.2	[28]
9	Ge	23	10	500	1.0–5.9	3.0–10	1.2	[28]
11	Ge	23	10	500	1.0–6.1	3.0–10	1.2	[28]
2	Si_0.2_Ge_0.8_	70	15	500	8.0	8.2	8	[42]
4	Si_0.2_Ge_0.8_	70	15	500	11	6.1	8	[42]
6	Si_0.2_Ge_0.8_	70	15	500	12	2.9	8	[42]
5.5	Si_0.03_Ge_0.93_Sn_0.04_	42	12	45	0.6–2.0	8.0–19	2	[43]
10	Si_0.017_Ge_0.927_Sn_0.056_	42	12	45	2.0–6.0	3.0–9.0	2	[43]]
8	Ge	15	15	500	2.4–5.2	13–18	1.6	[29]
8	Ge	48	27	1100	0.2–2.5	30–80	2	[This work]

All simulations are run using identical models with the inclusion of strain and composition‐dependent material properties for a random solid solution (explained in depth in Section 5). The difference in the two simulations is reflected in the alloy disorder scattering potential, $\Delta U_{\mathrm{alloy}}$. Random alloys can be treated as ordered, crystal structures using a technique called virtual crystal approximation (VCA) [44]. Under VCA, the lattice sites are occupied by pseudoatoms that interpolate between the behavior of component atoms. $\Delta U_{\mathrm{alloy}}$ captures the difference in valence band edge between the VCA and the disordered random alloy. In this study, it ignores any possibility of SRO. Consequently, $\Delta U_{\mathrm{alloy}}$ captures the strength of alloy disorder scattering experienced by carriers in the alloy system [45, 46, 47]. The values of $\Delta U_{\mathrm{alloy}}$ for current structures and previously reported structures are ${\Delta U}_{\mathrm{alloy}}^{\mathrm{curr.}}=0.8$ and ${\Delta U}_{\mathrm{alloy}}^{\mathrm{prev.}}=1.65$, respectively. The value of ${\Delta U}_{\mathrm{alloy}}^{\mathrm{curr.}}$ is determined using first‐principle calculations, and the value of ${\Delta U}_{\mathrm{alloy}}^{\mathrm{prev.}}$ is determined using statistical techniques described in Section 5.

The difference in $\Delta U_{\mathrm{alloy}}$ values for the two structures explains the difference in hole mobility, and indicates that our structure experiences smaller alloy disorder scattering compared to previously grown structures from the literature. This result is crucial because it explains that the difference in the strength of alloy disorder scattering is the reason behind the vastly different hole mobility values experienced by GeSn samples, despite nominally identical conditions like device structure (and hence strain), alloy compositions, doping, and operating conditions like applied voltage and temperature. $\Delta U_{\mathrm{alloy}}$ originates via the random occupation of alloy atoms in the crystal lattice. Furthermore, our results verify that the increased alloy disorder scattering in the other material offsets the mobility enhancement that would come from increased strain. It can be hypothesized that smaller values of $\Delta U_{\mathrm{alloy}}$ can be a result of deviation from a fully random crystal, indicating the exciting possibility of SRO.

It is important to note that a complete understanding of the physical reasons behind smaller alloy scattering is incomplete. Whether it is a consequence of SRO or some completely different phenomenon, requires a thorough structural analysis using techniques like APT, EXAFS, and 4DSTEM. However, the decisive impact of alloy disorder scattering on hole mobility and the possibility of achieving high hole mobility GeSn quantum wells are conclusively established. Subsequently, it is imperative to investigate the alloy disorder‐limited hole mobilities for the GeSn system. Referring back to Figure 2b, the dotted black line shows the estimated hole mobility for the current structure with 8% Sn, at 20K. It is seen that under similar conditions, even higher hole mobilities can be obtained. Currently, it can be predicted that this increase is two to threefold.

## 4 Conclusion

A new record‐high hole mobility of $8\times 10^{4}\;{\mathrm{cm}}^{2}\;V^{-1}\;s^{-1}$ has been achieved in shallow and undoped GeSn/Ge quantum well heterostructures grown using a commercial 200 mm growth process. The structural characterization reveals a high crystalline quality of the material. Furthermore, the large effective g‐factor (13.6) and small effective mass ($0.0689m_{0}$), consistent with previous studies, emphasize the material's potential for spintronic and fast spin qubit applications. Finally, with the theoretical study, we conclude that the high hole mobility values are a direct consequence of smaller alloy disorder scattering, indicating that GeSn systems with similar Sn compositions and strain can have a range of mobility values spanning an order of magnitude. We hypothesize that smaller values of $\Delta U_{\mathrm{alloy}}=$ 0.8 eV, compared to $\Delta U_{\mathrm{alloy}}$ = 1.65 eV for a typical random alloy [47, 48], can be a result of a deviation of the GeSn alloy from a truly random solid solution, indicating a possibility of SRO. The exact physical reason behind smaller alloy scattering and subsequent testing of SRO requires extensive structural study using techniques like APT [27, 43, 49], 4D‐STEM, EXAFS [26, 27], etc., which is beyond the scope of this paper. However, such a study would utilize the higher resolution of these techniques to assess the degree of atomic ordering and correlate, in addition to theoretical investigations, its affects on physical properties like mobility [43] or even spin qubit properties [29]. Additionally, even as we know that a variation in strain and alloy compositions changes the hole mobility, we predict that theoretically a two to three times higher hole mobility can be achieved *ceteris paribus*. This possibility of higher hole mobilities, in conjunction with existing properties of the GeSn system like SOC, further solidifies the potential of GeSn as a candidate for novel semiconductor technologies and high mobility electronic applications.

## 5 Methods

**FIGURE 7:**
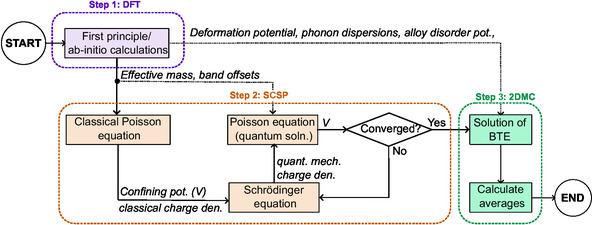
High‐level flowchart illustrating the theoretical model used in this study.

The three‐step simulation framework used in this study is shown in Figure 7. In summary, we use ab initio DFT calculations to extract basic material parameters needed for the next steps. Subsequently, we solve the 1D Schrödinger–Poisson (SCSP) problem to calculate the energy level structure. Finally, use the 2DMC method to solve the BTE for the quasi‐two‐dimensional hole gas (Q2DHG) that allows us to calculate the low‐field hole mobility ($\mu_{p}$).

### 5.1 Density Functional Theory

The DFT calculations are an important and necessary step in our simulation framework as they allow us to extract crucial material parameters. We use the Vienna ab initio simulation package [50] for this purpose. As suggested in previous work [30], we calculate the band structure of GeSn alloys using a special quasirandom structure [51] representation for random solid solution, using a 128‐atom simulation cell. We use local density approximations [52] with modified Becke–Johnson (mBJ) potential [53] and SOC as it is necessary for accurate representation of band structures of Ge and α‐Sn [23, 54, 55]. The calculated band structure is used to extract the energy bandgaps ($E_{g}$), the valence band offsets (VBOs), and the effective masses of holes ($m^{*}$) residing in the HH, LH, and SO bands. VBOs between a biaxially strained GeSn alloy and relaxed Ge are calculated similarly for different Sn concentrations and fit to an empirical model for the ease of implementation in the SCSP solver. The empirical expressions for VBO and $E_{g}$ are

(3) $$\mathrm{VBO}={\mathrm{VBM}}_{\mathrm{GeSn}}-{\mathrm{VBM}}_{\mathrm{Ge}}=2.01167x+0.00375$$

(4) $$E_{g}=0.742-\frac{4.8\times 10^{-4}\cdot T^{2}}{T+235}-1.74x+2.862x^{2}$$

for Sn composition *x*, and temperature T. VBM in Equation (3) stands for valence band maximum.

### 5.2 Self‐Consistent Schrödinger–Poisson System

We used the extracted DFT parameters in our in‐house SCSP solver to calculate the energy eigenstates ($E_{i}$) and the corresponding eigenfunctions ($\psi_{i}$), which, in turn, allow us to determine the sheet carrier densities of the occupied subbands. The solution of the Schrödinger wave equation is based on the parabolic band approximation for a spatially varying, anisotropic effective mass tensor. This is a reasonable assumption as the measured sheet carrier densities are relatively low, which suggests that carriers occupy states in the vicinity of the Γ‐point where nonparabolicity effects are not significant. The quantum–mechanically calculated charge density is used as input into the 1D Poisson equation, wherein we account for general Fermi–Dirac statistics, and a spatially varying dielectric constant ($\varepsilon_{r}$). A finite volume method with three‐point integration scheme and Newton–Raphson method is used for the solution of the 1D SCSP system [56, 57]. The system of equations for the 1D SCSP problem (assuming quantum confinement in z‐direction) are given below

(5) $$\begin{array}{r} \left\{ \begin{array}{l} \left(-\frac{\partial }{\partial z}\frac{1}{m_{⊥}(z)}\frac{\partial }{\partial z}+V_{\mathrm{conf}}(z)\right)\psi =E\psi \\ -\frac{\partial }{\partial z}\left(\varepsilon_{r}(z)\frac{\partial \varphi }{\partial z}\right)=q\rho_{\mathrm{qm}}\\ \rho_{\mathrm{qm}}=\frac{m_{∥}}{\pi \hbar^{2}}k_{B}T\sum_{i}\left(\ln\;\left[1+\exp\;\left(\frac{E_{f}-E_{i}}{k_{B}T}\right)\right]|\psi_{i}{|}^{2}\right)+F_{1/2}\left(\frac{E_{f}-E_{c}}{k_{B}T}\right) \end{array}\right. \end{array}$$

where φ is the electrostatic potential, $F_{1/2}(\cdot )$ is the Fermi integral of order half, $m_{⊥}$ is the effective mass perpendicular to surface (direction of confinement)$,m_{∥}$ is in‐plane effective mass (direction of free transport), and q is electron charge. The conduction band edge ($E_{c}$) and confining potential $V_{\mathrm{conf}.}$ capture the effect of VBOs. Other quantities are fundamental constants.

### 5.3 Two‐Dimensional Monte Carlo Method

The 2DMC method solves the BTE using path integral formalism [34]. It employs a free‐flight‐scatter scheme [58], wherein the carrier scattering rates are calculated within the first‐Born approximation (Fermi's golden rule) [35]. For quasi‐2D systems, calculation of scattering rates requires calculation of overlap integrals, which depend on the carrier wavefunctions extracted from the 1D SCSP system. The scattering mechanisms considered for the system are acoustic phonon, nonpolar optical phonon, surface roughness, and alloy disorder scattering. The scattering rates are calculated for all possible transitions between various subbands of HH, LH, and SO bands. We treat acoustic and optical phonons as bulk phonons.

The matrix elements for the relevant scattering between subbands n and m are given below.

#### 5.3.1 Acoustic Phonon Scattering

(6) $$|\left\langle n|U^{ac}(q)|m\right\rangle {|}^{2}=\frac{k_{B}T}{\rho \Omega \nu_{s}^{2}}\Xi_{ac}^{2}F_{\mathrm{nm}}$$

where the form factor $F_{\mathrm{nm}}$ is calculated using

(7) $$F_{\mathrm{nm}}=\int_{0}^{\infty }\psi_{n}^{2}(z)\psi_{m}^{2}(z)dz$$

$\Xi_{ac}$ is the acoustic phonon deformation potential derived from first principles calculations, Ω is the volume, and ρ and $v_{s}$ are the density and speed of sound in the material.

#### 5.3.2 Non‐Polar Optical Phonon Scattering

(8) $$|\left\langle n|U^{op}(q)|m\right\rangle {|}^{2}=\frac{\hbar \;\Xi_{o}^{2}}{2\rho V\omega_{o}}F_{\mathrm{nm}}$$

where $\Xi_{o}$ is the nonpolar optical phonons deformation potential and $\omega_{0}$ is the phonon frequency.

#### 5.3.3 Surface Roughness Scattering

The general form of the matrix element for surface (interface) roughness scattering is [59],

(9) $$|\left\langle n|U^{sr}(q)|m\right\rangle {|}^{2}=S(q)\Gamma_{nm}^{2}$$

where S(q) is the power spectral density, which is a Fourier transform of the autocorrelation function that characterizes the roughness of the heterointerface, and $\Gamma_{nm}$ is calculated using

(10) $$\Gamma_{nm}=\frac{\hbar^{2}}{2m^{*}}\frac{{d\psi }_{n}}{dz}\frac{{d\psi }_{m}}{dz}{|}_{z=0}=\int_{0}^{\infty }\left(\psi_{n}(z)\frac{\partial V_{sr}(z)}{\partial_{z}}\psi_{m}(z)-E_{m}\frac{{d\psi }_{n}}{dz}\psi_{m}(z)+E_{n}\psi_{n}(z)\frac{{d\psi }_{m}}{dz}\right)dz$$

where $V_{sr}(z)$ is the spatially varying potential energy term [60]. For a Gaussian autocorrelation function [61, 62, 63], the power spectral density is also Gaussian and is given by,

(11) $$S_{G}(q)=\pi \Delta^{2}L^{2}\exp\;(-\frac{q^{2}L^{2}}{4})$$

Parameters Δ and L are the root mean squared (r.m.s.) height of the roughness and the roughness correlation length, respectively. Extensive analysis is done by Goodnick et al [61] with high‐resolution transmission electron microscopy measurements, to validate the assumption of Gaussian correlation. They found that exponential correlation describes roughness much better than Gaussian correlation, irrespective of the growth conditions. Roughly speaking, it means that the interface may be regarded as consisting of terraces of a few nanometers in size separated by atomic steps of a few tenths of a nanometer. This result has also been confirmed by atomic force microscope measurements [64]. The power spectral density for the exponentially correlated roughness is,

(12) $$S_{E}(q)=\frac{\pi \Delta^{2}L^{2}}{{\left(1,+,q^{2},L^{2},/,2\right)}^{3/2}}$$

#### 5.3.4 Alloy Disorder Scattering

(13) $$|\left\langle n|U^{alloy}(q)|m\right\rangle {|}^{2}=\frac{x(1-x)a_{0}^{3}}{8V}{\left(\Delta ,U_{\mathrm{alloy}}\right)}^{2}F_{\mathrm{nm}}$$

where $\Delta U_{\mathrm{alloy}}$ is the alloy disorder scattering potential and $a_{0}$ is the lattice constant.

Mobility limited by a few other scattering mechanisms including background impurities, surface states, etc., can be estimated analytically as discussed by Monroe et al. [65]. A detailed description of these mechanisms, and analysis using additional SIMS data, is provided in the SI. For a background impurity density of $1\times 10^{15}\;{\mathrm{cm}}^{-3}$, around the detection limit of phosphorous for SIMS data, the limiting value of mobility is estimated as $2.84\times 10^{5}\;{\mathrm{cm}}^{2}\;V^{-1}\;s^{-1}$. For a remote impurity sheet density of $1\times 10^{12}\;{\mathrm{cm}}^{-2}$, a value consistent with either unintentional dopants or interface traps, the limiting value of mobility is estimated as $7.86\times 10^{5}\;{\mathrm{cm}}^{2}\;V^{-1}\;s^{-1}$. For reg1 where a single channel is present, the limiting value of mobility due to surface roughness is around $1.2\times 10^{6}\;{\mathrm{cm}}^{2}\;V^{-1}\;s^{-1}$. Consequently, these scattering mechanisms do not play a significant role in determining the hole mobilities of our structures at low temperatures. Important parameters used to calculate the scattering rates are shown in Table 2.

**TABLE 2:** Material parameters used for calculating hole scattering rates for 2DMC.

Parameter (symbol)	Value	Description
$\varepsilon_{r}$	16.2	Dielectric constant
$m_{HH}^{*}$ Ge(100)	0.232 $m_{0}$	Effective mass of holes in HH band in unstrained Ge
$m_{LH}^{*}$ Ge(100)	0.059 $m_{0}$	Effective mass of holes in LH band in unstrained Ge
$m_{SO}^{*}$ Ge(100)	0.116 $m_{0}$	Effective mass of holes in SO band in unstrained Ge
$\Xi_{ac}$	4.6 eV	Acoustic deformation potential for holes
ρ	$5232\;\mathrm{kg}/m^{3}$	Crystal density
$v_{s}$	6310 m/sec	Speed of sound
$\Xi_{op}$	$8\times 10^{10}$ eV	Optical deformation potential
$\hbar \omega_{o}$	37 meV	Optical phonon energy
Δ	$2.84\times 10^{-10}\;m^{-1}$	r.m.s. height of roughness
L	$24.2\times 10^{-10}\;m^{-1}$	Roughness correlation length
${\Delta U}_{\mathrm{alloy}}^{\mathrm{prev.}}$	1.65 eV	Alloy disorder scattering potential for structures from literature [28]
${\Delta U}_{\mathrm{alloy}}^{\mathrm{curr.}}$	0.8 eV	Alloy disorder scattering potential used in this study

### 5.4 Statistical Methods

The free‐flight‐scatter scheme described above is a stochastic method. Free flight, as the name suggests, is the unimpeded transport of the carriers under the influence of an electric field (E). This free flight is impeded via different lattice phenomena, which are captured as different scattering mechanisms. The interplay between these two phenomena culminates in a situation wherein the carrier ensemble moves along the electric field at an average velocity referred to as drift velocity ($v_{d}$). The values of $v_{d}$ are obtained as a result of the Monte Carlo simulations. The free‐flight times and different scattering mechanisms are chosen randomly, in accordance with their probabilities of occurrence. These are determined by the scattering rates described in the previous section.

The MC simulations are run for M batches, each with N particles. Carrier mobilities are dependent on the electric fields experienced by the particles. The net electric field experienced by carriers is a sum of the globally applied electric field ($E_{0}$) and local electric fields due to charge distribution. This effect becomes important for low‐field transport. To eliminate the effect of local fluctuations, we run each batch of simulations for an electric field given by $E_{j}=E_{0}(1+r_{j})$, where $r_{j}$ is a random number sampled from a uniform distribution between −0.1 and 0.1. This ensures that the average electric field experienced by the particles, $\bar{E}=E_{0}$. Consequently, the carrier drift velocities are given by,

(14) $$\begin{array}{r} {\bar{v_{d}}}_{,j}=\frac{1}{N}\overset{N}{\underset{i=1}{\sum }}v_{d,ij} \end{array}$$

and the carrier mobility is given by,

(15) $$\begin{array}{r} \mu =\frac{1}{ME_{0}}\sum_{j=1}^{M}\frac{{\overset{―}{v_{d}}}_{,j}}{1+r_{j}} \end{array}$$

It should be noted that for constant E, $r_{j}=0$, and Equation (15) collapses into the general expression $\mu =v_{d}/E$. The variance in mobility is given by,



(16)

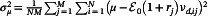




The error in simulated mobility w.r.t. experimentally measured mobility is given by

(17) $$\begin{array}{r} \varepsilon_{\mu }=\frac{\left|\mu -\mu_{\mathrm{expt}.}\right|}{\mu_{\mathrm{expt}.}}\times 100 \end{array}$$

In this study, we observe that the nominal value of $\Delta U_{\mathrm{alloy}}$, which could estimate the hole mobility of structures from the literature (refer to Figure 6), underestimated the hole mobility values observed in this study, likely due to the overcontribution of alloy disorder scattering. Consequently, we ran preliminary simulations to determine the value of $\Delta U_{\mathrm{alloy}}$ that verifies the experimentally observed mobility values. If $\mu_{\mathrm{expt}.}$ represents experimentally measured hole mobility, and $\hat{\mu }$ represents simulated hole mobility, then the value of $\Delta U_{\mathrm{alloy}}$ is estimated using the minimization problem,

(18) $$\begin{array}{r} \min_{\Delta U_{\mathrm{alloy}}\in (0,2)}||\hat{\mu }-\mu_{\mathrm{expt}.}|{|}^{2} \end{array}$$

2DMC is a very noisy process due to multiple intersubband and intrasubband scattering mechanisms. A large number of particles are needed to reduce the variance in simulated data. Our calculations are performed for $M=50,N=10^{5}$ for each temperature and applied bias. We obtained results within 95% accuracy w.r.t. the experimental data, and a max variance of about 4876.36 for simulations at 1K.

Each magnetotransport measurement, classical and quantum Hall measurements, was performed with a different Hall bar device (*n* = 1 sample size) from a $1{\mathrm{cm}}^{2}$ die with 20 devices. A second round of fabrication independently verified the record‐high mobility for the material. The gate voltage‐dependent density‐mobility data are preprocessed by thresholding the data with $R^{2}=0.99$ from the linear fits. Mobility–density data, in both measurements, is calculated from a magnetic field range from −0.5 to 0.5 T.

## Supporting Information

Additional supporting information can be found online in the Supporting Information section. **Supporting Figure S1**: (a) Scanning electron microscopy image of the cross‐sectioned Hall bar device showing the extraction site. (b) Integrated EDS line scan data overlayed on a HAADF micrograph. (c) Integrated EDS line scan showing adjusted counts versus distance for Pt (red), Ti (blue), O (green), Ge (magenta), and Sn (yellow). **Supporting Figure S2**: Ge concentration profiles derived from SIMS data fitted with an error function for (a) the top barrier/well interface and (b) the bottom barrier/well interface. **Supporting Figure S3**: SIMS depth profiles showing the spatial distribution of background impurities P (blue) and O (green) overlaid with the Ge atomic percentage (red). **Supporting Figure S4**: Magnetotransport data for the density of 2.2 × 10^11^ cm^−2^ showing longitudinal resistance (blue, left axis) and normalized quantum Hall resistance (orange, right axis) versus magnetic field. **Supporting Figure S5**: Temperature dependence of the longitudinal resistance Shubnikov–de Haas oscillations for the density of 2.2 × 10^11^ cm^−2^ plotted as a function of Landau levels. **Supporting Figure S6**: Arrhenius plot of the longitudinal resistance for the *ν* = 3 Landau level minimum versus inverse temperature. The linear fit allows for the extraction of the thermal excitation gap.

## Funding

The authors have nothing to report.

## Supporting information

Supplementary Material

## Data Availability

The data that support the findings of this study are available from the corresponding author upon reasonable request.
