# Supplementary material for: Experimental Characterization and Modeling of High Hole Mobility GeSn Quantum Wells: The Role of Alloy Disorder Scattering
Source: Small Sci. 2026 Mar 31;6(4):e202500589. doi: 10.1002/smsc.202500589 (PMC13154920; doi:10.1002/smsc.202500589)
Supplement: Supplementary file 1 — Supplementary Material [file SMSC-6-e202500589-s001.pdf]

Supplemental Information — Experimental Characterization and Modeling of High Hole Mobility GeSn Quantum Wells: The Role of Alloy Disorder Scattering

Troy A. Hutchins-Delgado Siddhant Gangwal* Steven Akwabli Adelaide Bradicich Priyanka Petluru Hryhorii Stanchu Sudip Acharya Robin Scott Nick Rosson Michael Povolotskyi Chia-Tse Tai Chia-You Liu Jiun-Yun Li Michael P. Lilly Winson C.H. Kuo Stephen D. House Dragica Vasileska Shui-Qing Yu Tzu-Ming Lu*

** These authors contributed equally to this work.*

1 Additional Structural Analysis

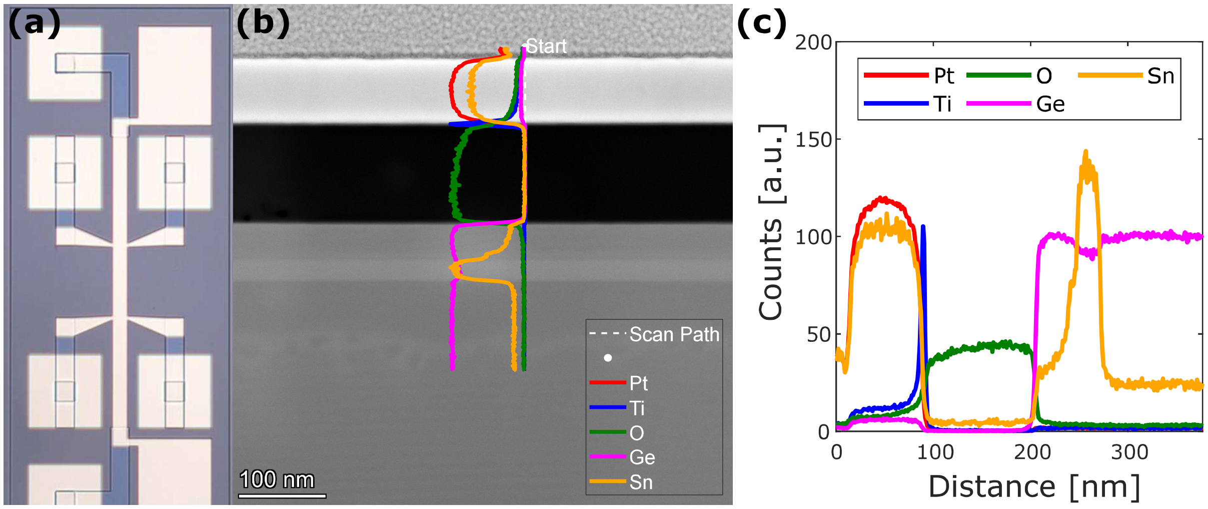

Figure S1: (a) SEM image of the cross-sectioned Hall bar device showing the extraction site. (b) Integrated EDS line scan data overlayed on a HAADF micrograph. (c) Integrated EDS line scan showing adjusted counts versus distance for Pt (red), Ti (blue), O (green), Ge (magenta), and Sn (yellow).

Compositional information about the studied structure was obtained by both energy dispersive x-ray spectroscopy (EDS) and secondary ion mass spectrometry (SIMS) as mentioned in the main text. Cross-sectional lift-outs for nanoscale structural and compositional characterization were prepared using a Thermo Fisher Scientific Scios 2 focused ion beam/secondary electron microscope (FIB/SEM). Samples were extracted from a Hall bar device (Figure S1(a)) where both the gate stack and Ohmic stacks could be analyzed. These samples were subsequently examined with a Thermo Fisher Scientific Titan ChemiSTEM probe-aberration-corrected S/TEM equipped with a 0.7 sr Super-X energy dispersive X-ray spectroscopy (EDS) system. The analysis was performed at an operating voltage of 200 kV and probe current of 150 pA.
The EDS data is summarized in Figures S1(b)-(c), whereby Figure S1(b) shows an integrated line scan of the EDS data overlayed with a high-angle annular dark field (HAADF) micrograph and Figure S1(c) focuses on the integrated line scan data showing the EDS counts (adjusted) versus distance. The EDS line scans highlight the following elements: Pt (red), Ti (blue), O (green), Ge (magenta), and Sn (yellow). For clarity, the EDS counts were respectively scaled as follows: x0.5, x2.0, x2.0, x1.0, and x10.0. While we extracted atomic concentration estimates from the EDS data (which we used for our simulations), we also elected to perform SIMS analysis for its greater depth resolution to provide a better structural analysis (as shown in the main text). Figure S2 shows the Ge concentration from the SIMS data fit with an error function, $a + b * erf((x - 40) * c)$, to provide a degree of sharpness of the quantum

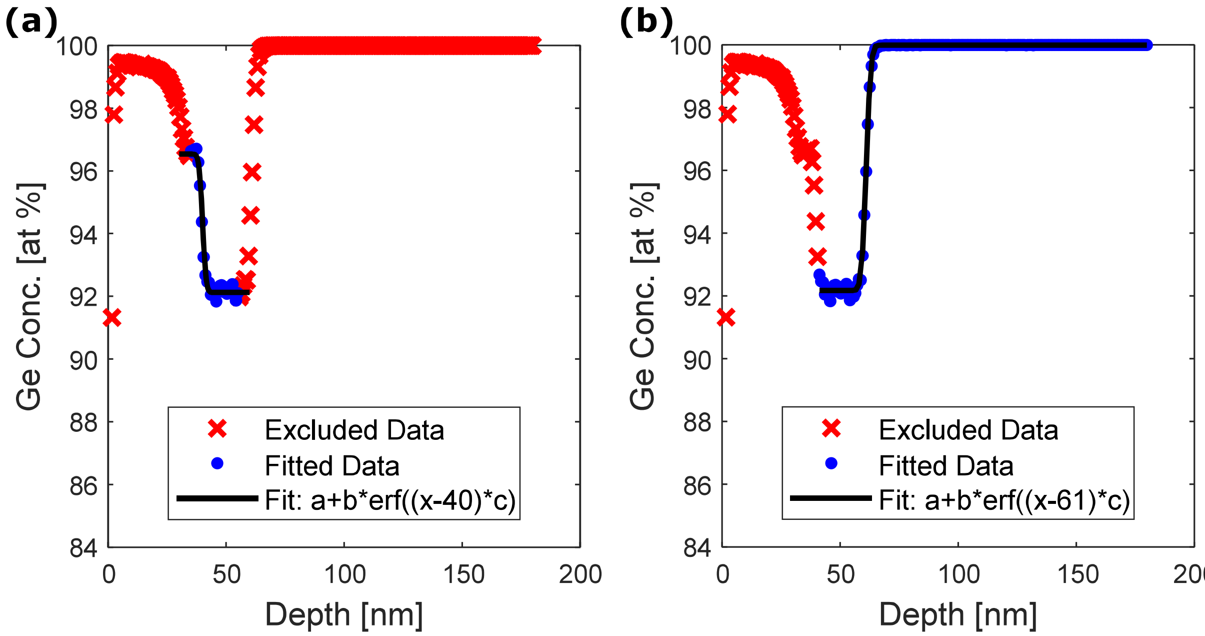

Figure S2: Ge concentration profiles derived from SIMS data fitted with an error function for (a) the top barrier/well interface and (b) the bottom barrier/well interface.

well interfaces. A more physical form of the fitting model is [1]:

$$x = \frac{1}{2}(x_w + x_b) + \frac{1}{2}(x_w + x_b) \cdot erf(\frac{d - d_0}{\sqrt{2}\lambda}) \quad (1)$$

Where λ is the characteristic length, d_0 is the transition distance, and x_w and x_b are the concentrations in the well and barrier, respectively. It is clear to see that the characteristic length is simply inversely proportional to the fitting constant c with a multiplicative factor. We extracted characteristic lengths of (1.20 ± 0.28) nm and (1.55 ± 0.06) nm for the top barrier/well interface (Figure S2(a)) and the bottom barrier/well interface (Figure S2(b)), respectively. Here, the error corresponds to the standard deviation calculated from the 95% confidence interval of the fit. These values, while not extraordinary, are respectable values for a high-quality structure.

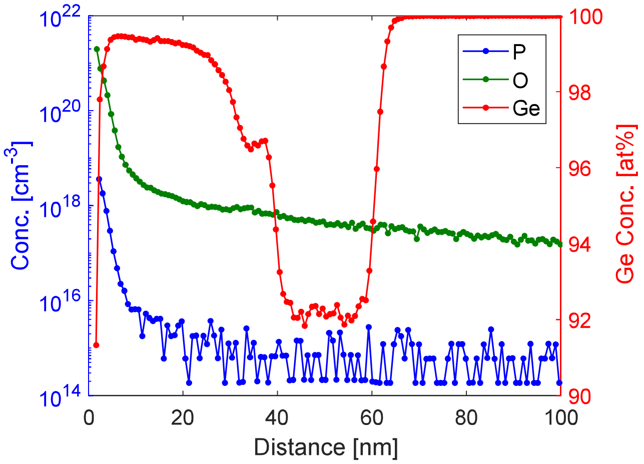

Figure S3: SIMS depth profiles showing the spatial distribution of background impurities P (blue) and O (green) overlaid with the Ge atomic percentage (red).

Along with the atomic concentrations of the structure, SIMS analysis also provided data for the common impurities. Figure S3 shows the overlay of concentrations of P (blue) and O (green) with the Ge atomic percentage (red) vs distance to provide the spatial profile of the impurities. Here, P is of particular interest because, if ionized, it would act as charged scattering center and would have an impact on

the mobility-density relation (main text). We estimated a remote impurity scattering limit, μ_{remote}, of $7.85 \times 10^5\,\mathrm{cm^2\,V^{-1}\,s^{-1}}$ using the following equation [2]:

$$\mu_{remote} \approx 16\pi^{1/2} g_v^{1/2} g_s^{1/2} e n_s^{3/2} h_{eff}^3 / \hbar N_\square \tag{2}$$

Here, $g_v = 1$ and $g_s = 2$ are valley and spin degeneracies, respectively. Also, n_s is the experimental sheet density of $2.2 \times 10^{11}\,\mathrm{cm^{-2}}$ while $N_\square$ is a remote surface charge density of $1.0 \times 10^{12}\,\mathrm{cm^{-2}}$. A very conservative value of 50 nm was used for the effective distance h_{eff}. Likewise, it is seen that the remote surface charge density is a conservative value closer to that of charges from interface traps than charged impurities. A more reasonable mobility limit from background impurities, μ_{3D}, is estimated from the SIMS data and calculated from the equation [2]:

$$\mu_{3D} \approx (g_v^{3/2} g_s^{3/2} / 4\pi^{1/2})(e n_s^{1/2} / \hbar N_{3D}) \tag{3}$$

Here, we used the background impurity density of $1.0 \times 10^{15}\,\mathrm{cm^{-3}}$ and obtained a mobility limit of $2.84 \times 10^5\,\mathrm{cm^2\,V^{-1}\,s^{-1}}$. With these mobility limit estimates, it is now clear to see (in the main text) that the mobility limit in the low-density regime is due to alloy disorder scattering as we concluded. More importantly, while our results show a four-fold improvement over the previous high-mobility record because of a reduced alloy scattering potential, it may be possible to gain at least another four-fold improvement in mobility in the background impurity limit. An improvement of this magnitude would make GeSn/Ge more competitive as material platform for spin qubits on par with Si/SiGe and Ge/SiGe.

2 Additional Details on Quantum Hall Analysis

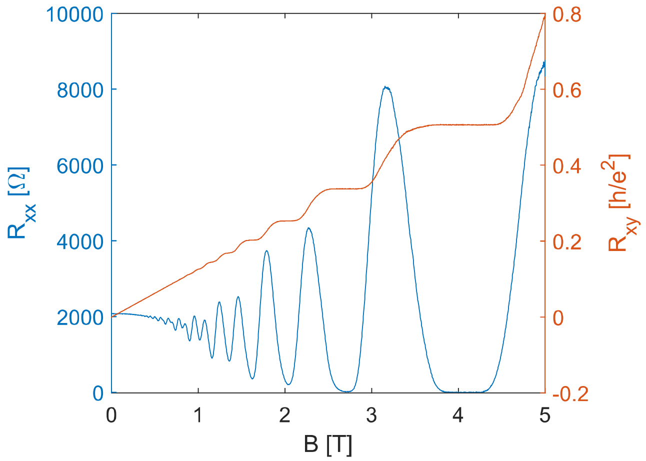

Figure S4: Magnetotransport data for the density of $2.2 \times 10^{11}\,\mathrm{cm^{-2}}$ showing longitudinal resistance (blue, left axis) and normalized quantum Hall resistance (orange, right axis) versus magnetic field.

Analysis of Shubnikov-de Hass (SdH) oscillations from integer quantum Hall measurements allows extraction of effective g-factor and effective mass from the temperature dependence of the longitudinal resistance minima of each integer Landau level ν. The following analysis follows that of Lodari *et. al.* [3] and is summarized by Figures S4-6. Figure S4 shows the longitudinal resistance data in blue (left) and the transverse/Hall resistance data normalized by the resistance quantum in orange (right). Here, the minima in the SdH oscillations correspond to the plateaus in the Hall resistance whereby the value of the Hall resistance (in quantum units) is the inverse of the integer Landau level.
The SdH oscillations can be represented instead as a function of Landau Levels with the conversion relation being defined as:

$$\nu_{3D} = \frac{n_s}{N_\phi} = \frac{e}{h}\frac{n_s}{B_\perp} \tag{4}$$

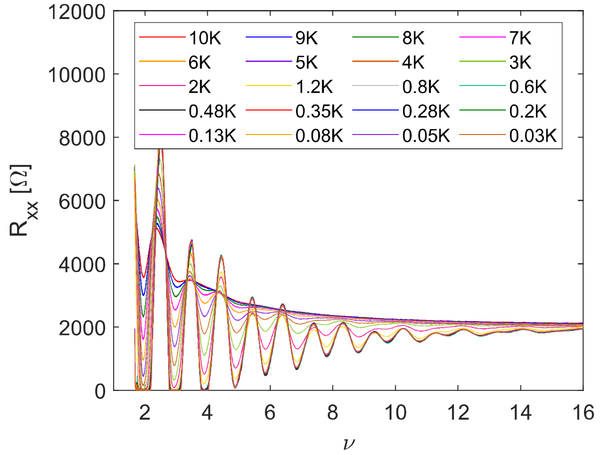

Figure S5: Temperature dependence of the longitudinal resistance Shubnikov-de Haas oscillations for the density of $2.2 \times 10^{11}\,\mathrm{cm}^{-2}$ plotted as a function of Landau levels.

Whereby the magnetic flux number density, N_ϕ, is defined by perpendicular magnetic field, $B_\perp$, multiplied by a factor of e/h and n_s is the sheet density. Figure S5 shows the temperature dependence of the SdH oscillations of the longitudinal resistance as a function of the Landau levels. Here, the sheet density is directly obtained from the linear portion of the Hall resistance slope which is $2.2 \times 10^{11}\,\mathrm{cm}^{-2}$. The density could instead be calculated by performing a linear fit using the Landau level/magnetic field conversion relation. The assumption here is that the SdH oscillations are due to a sheet density of a single channel because there is no beating present. As we showed in the main text, there is a second channel at the surface, but its mobility is so low that it does not contribute to the SdH oscillations. However, this still creates a slight discrepancy between the densities obtained from the two methods. This can be seen in Figure S5 where the minima are not exactly at integer values but are very close and the values are still verified by the quantum Hall plot of Figure S4. Also, the slight discrepancy has no effect on the temperature dependence which is of interest for the thermal excitation gap analysis. In the main text we only showed temperatures of 0.8 K, 1.2 K, 2 K, 3 K, 4 K for clarity as these are temperatures that show significant changes of the minima values for most of the Landau levels. However, the full experiment was for 20 temperatures ranging from 30 mK to 10 K which is fully represented in Figure S4.

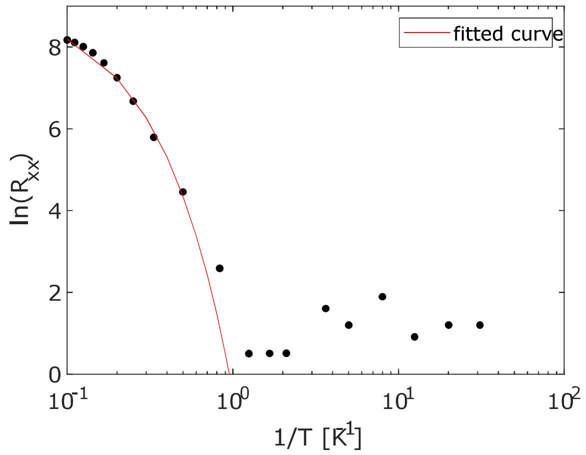

Figure S6: Arrhenius plot of the longitudinal resistance for the $\nu = 3$ Landau level minimum versus inverse temperature. The linear fit allows for the extraction of the thermal excitation gap.

A thermal excitation gap analysis of each Landau level can be performed by performing a linear fit between the natural log of the longitudinal resistance at the minima and inverse temperature due to the following relation arising from Boltzmann's statistics:

$$ln(R_{xx,\nu}) \propto -\frac{\Delta_\nu}{2k_B T} \tag{5}$$

With Δ_ν representing the thermal excitation gap. Figure S6 shows the corresponding Arrhenius plot of the $\nu = 3$ data along with its fit for extracting its thermal excitation gap. With all the thermal excitation gaps extracted, the effective g-factor and effective mass can be extracted from the magnetic field dependence as follows:

$$\Delta_\nu^{odd} = g^* \mu_B B \tag{6}$$

$$\Delta_\nu^{even} = \frac{he}{m^*} B - g^* \mu_B B \tag{7}$$

with μ_B being the Bohr magneton and g^* and m^* being the effective g-factor and effective mass, respectively. These are the equations we used in the analysis in the main text and are used as fit lines of Figure 5.

References

[1] D. Zhang, J. Lu, Z. Liu, F. Wan, X. Liu, Y. Pang, Y. Zhu, , et al., *Applied Physics Letters* **2022**, *121*, 2 022102.

[2] D. Monroe, Y. Xie, E. Fitzgerald, P. Silverman, G. Watson, *Journal of Vacuum Science & Technology B: Microelectronics and Nanometer Structures Processing, Measurement, and Phenomena* **1993**, *11*, 4 1731.

[3] M. Lodari, O. Kong, M. Rendell, A. Tosato, A. Sammak, M. Veldhorst, A. R. Hamilton, G. Scappucci, *Applied Physics Letters* **2022**, *120*, 12 122104.
